# Barriers to public engagement with biodiversity conservation

**DOI:** 10.1111/cobi.70078

**Published:** 2025-07-13

**Authors:** Emily A. Gregg, Georgia E. Garrard, Sarah A. Bekessy, Alexander M. Kusmanoff, Jen K. Martin, Matthew J. Selinske, Lindall R. Kidd, Jennifer A. Robinson

**Affiliations:** ^1^ ICON Science Research Group, School of Global, Urban, and Social Studies RMIT University Melbourne Victoria Australia; ^2^ School of Agriculture, Food and Ecosystem Sciences The University of Melbourne Melbourne Victoria Australia; ^3^ School of BioSciences The University of Melbourne Melbourne Victoria Australia; ^4^ School of Media and Communication RMIT University Melbourne Victoria Australia

**Keywords:** audiences, behavior, communications, community‐based conservation, comportamiento, comunicación, conservación basada en la comunidad, público, 传播, 基于社区的保护, 受众, 行为

## Abstract

Addressing biodiversity loss requires public engagement and action, including changes to individual consumption habits, support for on‐the‐ground conservation actions, and advocacy for government action and policy change. Conservation organizations are increasingly focused on encouraging probiodiversity attitudes and behaviors through interventions, such as education programs and marketing campaigns. Yet, motivating public audiences to change their behavior or become more active participants in conservation remains a challenge. We used a strategic communication approach to conceptualize barriers to public engagement with conservation and explored how these barriers manifest differently across audiences based on their current level of engagement (e.g., aware vs. active). The psychological, social, and structural barriers discussed are lack of knowledge, misaligned values, low self‐efficacy, low personal or social relevance, and limiting structural context. These barriers are recognized across conservation science, behavioral science, and social change literature. Many different communication approaches may be used to overcome these barriers, including raising awareness, working with values, social norming, strategic calls to action, social mobilization, and advocacy. Regardless of the approach, understanding individual behaviors, audience types, and their social context is key to supporting biodiversity conservation actions and positive social change.

## Abstract (es)

Barreras para la participación pública en la conservación de la biodiversidad

**Resumen:**

Para enfrentar la pérdida de biodiversidad se necesita el compromiso y la acción del público, lo que incluye cambios en los hábitos de consumo individuales, el apoyo a las acciones de conservación sobre el terreno y la defensa de la acción gubernamental y el cambio de políticas. Las organizaciones conservacionistas se centran cada vez más en fomentar actitudes y comportamientos favorables a la biodiversidad mediante intervenciones como programas educativos y campañas de marketing. Sin embargo, motivar al público para que cambie su comportamiento o participe más activamente en la conservación sigue siendo un reto. Utilizamos un enfoque de comunicación estratégica para conceptualizar las barreras que dificultan el compromiso del público con la conservación y estudiamos cómo estas barreras se manifiestan de forma diferente según el público y su nivel de compromiso actual (por ejemplo, consciente o activo). Las barreras psicológicas, sociales y estructurales analizadas son la falta de conocimientos, los valores desalineados, la baja autoeficacia, la escasa relevancia personal o social y el contexto estructural limitante. Estas barreras están reconocidas en las ciencias de la conservación, las ciencias del comportamiento y la literatura sobre el cambio social. Para superar estas barreras se pueden utilizar muchos enfoques de comunicación diferentes, como la sensibilización, el trabajo con valores, la creación de normas sociales, las llamadas estratégicas a la acción, la movilización social y la promoción. Los comportamientos individuales, el público y el contexto social pueden trabajar juntos para apoyar las acciones de conservación de la biodiversidad y el cambio social positivo.

## 公众参与生物多样性保护的障碍

**【摘要】** 解决生物多样性丧失的问题需要公众参与和公众行动, 这包括改变个人消费习惯、支持实地保护行动, 以及倡导政府行动和政策变革。保护组织越来越重视通过教育计划和营销活动等干预措施, 来鼓励人们采取有利于生物多样性的态度和行为。然而, 激励公众改变其行为或更积极地参与保护行动仍是一项挑战。本研究利用一种战略传播方法, 将公众参与保护的障碍概念化, 并探讨了这些障碍如何在不同参与程度的受众(如有保护意识相较于积极参与)中表现出差异。我们讨论的心理、社会和结构性障碍包括知识缺乏、价值观不一致、自我效能低、私人或社会关联性低, 以及结构性背景限制。这些障碍在保护科学、行为科学和社会变革文献都得到了认可。我们提议, 可采取多种不同的传播方式来克服这些障碍, 包括提高意识、结合价值观开展工作、塑造社会规范、提出有战略性的行动号召、社会动员及宣传。个人行为、公众和社会环境可共同支持生物多样性保护行动和积极的社会变革。**【翻译:胡怡思;审校:聂永刚】**

## INTRODUCTION

As the major driver of biodiversity loss, human behavior presents both an opportunity and a challenge for conservation practitioners (Clayton et al., 2013; Schultz, 2011). In biodiversity conservation research and practice, there is increasing interest in implementing strategic and targeted programs to raise awareness or concern, incentivize probiodiversity behaviors, or motivate greater social and political change (Reddy et al., 2017). Traditional knowledge‐deficit approaches are increasingly replaced by more sophisticated approaches from behavioral science, psychology, political science, cultural studies, communication, and sociology (Clayton & Myers, 2015; Green et al., 2019; Nisbet & Scheufele, 2009; Reincke et al., 2020; Selinske et al., 2018). Among these, social marketing is particularly well established in biodiversity conservation (Selinske et al., 2021, 2020; Veríssimo, 2013, 2019; Veríssimo et al., 2017). Most of these approaches recognize that understanding the broader social context of a behavior is key to setting feasible and effective objectives and strategies to achieve them. However, not all explicitly incorporate this social context in their frameworks, potentially limiting understanding of different social and behavioral change approaches and levers.

### Systems models for social and behavioral change

Numerous approaches can be applied to understand engagement with biodiversity conservation (e.g., COM‐B model [Michie et al., 2011]). Systems‐based models allow for visibility of a wider range of outcomes and levers, such as social mobilization and advocacy. The United Nations Children's Fund Social and Behavior Change (SBC) approach systematically seeks to change sociocultural context and individual behaviors through 3 types of action: advocacy; social mobilization and behavior change communication; and use of channels, such as interpersonal communication, community or folk media, mass media, and digital or social media (Petit, 2019; UNICEF, 2024). The SBC approach has been applied to wildlife conservation in combination with a behavioral economics approach (e.g., Change Wildlife Consumers [changewildlifeconsumers.org]), which is in strong contrast to knowledge‐deficit approaches.

**FIGURE 1:**
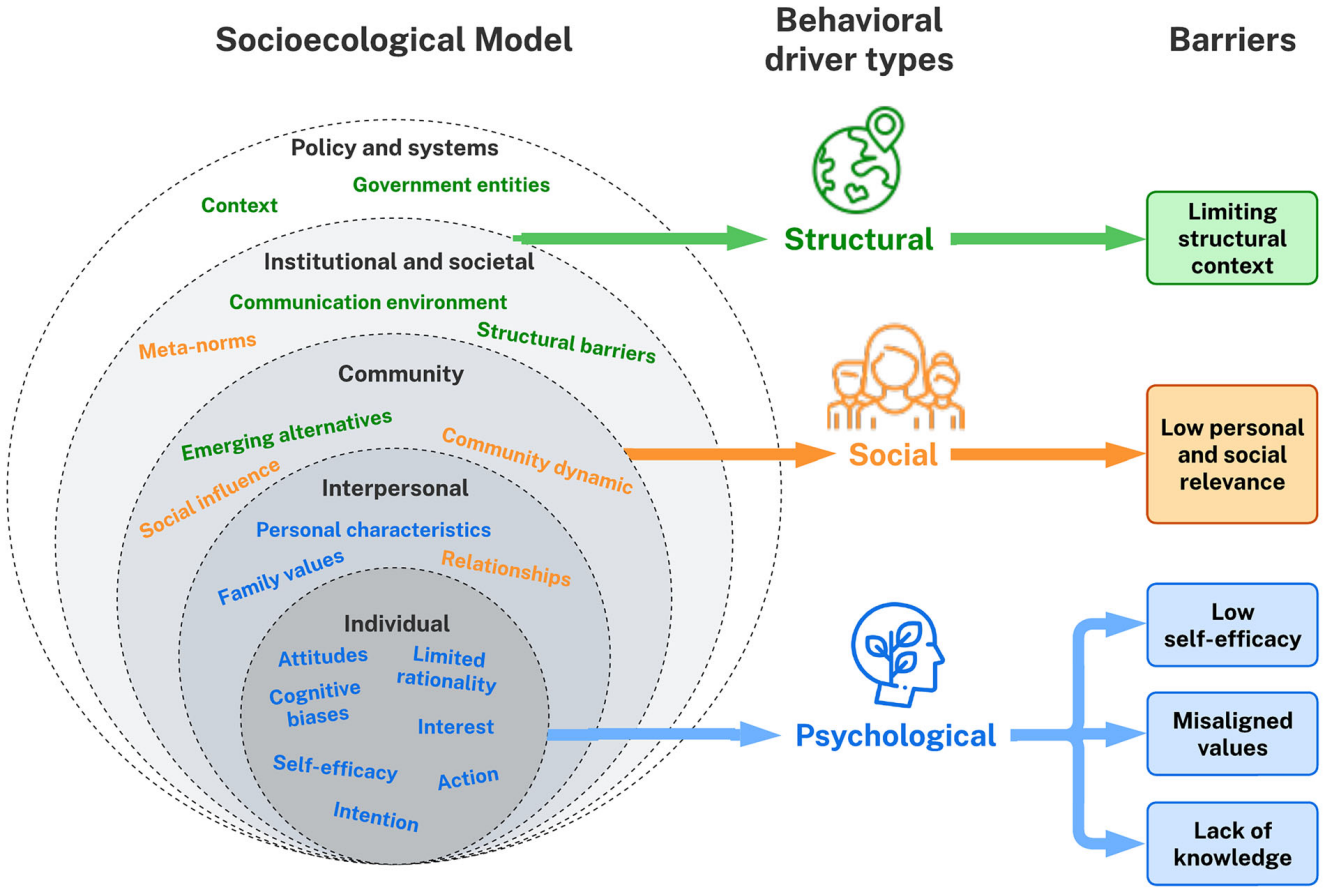
Socioecological model populated with the behavioral drivers model's structural (green), social (orange), and psychological (blue) behavioral drivers and linked to the structural, social, and psychological barriers discussed in text. Figure adapted from Petit (2019, Image 25, p. 53).

The SBC approach uses 2 foundational models: socioecological model (SEM) (Bronfenbrenner, 1977) and the behavioral drivers model (Petit, 2019) (Figure 1). The SEM provides a framework for considering how behavior can be influenced through activities at different levels of the sociocultural context: individual, interpersonal (family or peer), community, institutional and societal, and policy and systems. The SEM is multidirectional: each level can influence all others. For example, although ecotourism organizations (institutional and societal) may discourage human–wildlife contact during wildlife experiences (individual), posting selfies on social media can influence not only peers (interpersonal) but also online networks (community), potentially encouraging the behavior at a larger scale (Otsuka & Yamakoshi, 2020). The behavioral drivers model explores why people do what they do by describing psychological (e.g., attitudes), social (e.g., social influence), and structural (e.g., government entities) drivers of behavior (Petit, 2019). These drivers can be visualized in the SEM, enabling a holistic representation of the complexity of social and behavioral change (Figure 1).

### Barriers to social and behavioral change

Drivers and barriers can motivate or demotivate individuals to adopt a behavior. Barriers prevent or increase the difficulty of an individual actioning a behavior (Breakthrough ACTION, 2017) and can manifest at psychological, social, and structural levels (McKenzie‐Mohr, 2011) (Figure 1). Understanding the barriers to engagement is a key aspect of planning and designing communication approaches (McKenzie‐Mohr, 2011; Nilsson et al., 2020; White & Trower, 2021). Previous work has identified barriers to proenvironmental behavior in the context of energy conservation, water conservation, and climate change (Gifford, 2011; Han & Hyun, 2016; Kollmuss & Agyeman, 2002; Lacroix & Gifford, 2017; Lacroix et al., 2019). Barriers identified in the context of climate change (Lacroix et al., 2019) have also been demonstrated in the fields of biodiversity conservation (Bosone et al., 2022). Namely, these barriers are lack of knowledge, perceptions of change being unnecessary or ineffective, conflicting goals and aspirations, tokenism, interpersonal relations, and external attribution of responsibility to act.

In our experience, 2 of the most common questions posed by our conservationist colleagues are as follows: why are people not doing anything and how does one get people engaged? We present a framework to help answer both questions. Understanding barriers will help conservation professionals better understand their audiences and the social and behavioral change approaches that can be used to overcome barriers to engagement. To explore how to segment audiences for effective communication approaches when people are highly individual, we drew on a theory, the situational theory of problem solving, that examines how people respond based on their relationship to the issue.

### Situational theory of problem solving

**FIGURE 2:**
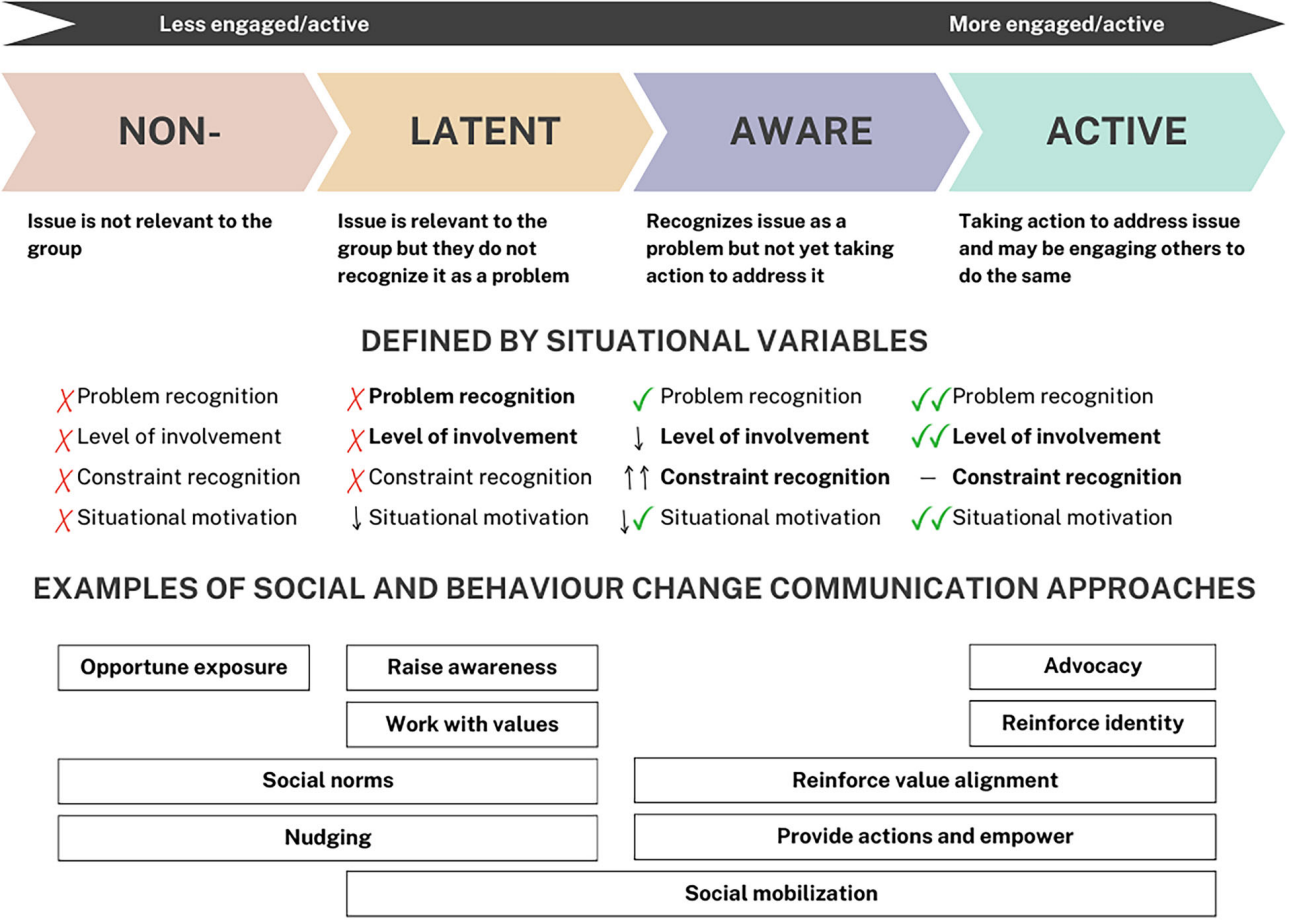
Conceptualization of the 4 publics defined by the situational theory of problem solving (Kim & Grunig, 2011), the situational variables they are defined by, and examples of communication approaches suited to these publics (bold, situational variables that are typical targets for influence from communication approaches; icons, how situational variables are expressed; red cross, not present; down arrow, low; dash, no clear expression; up arrow, high; check mark, present; multiple icons express varying expression or extent).

The situational theory of problem solving is a strategic communication theory used to conceptualize how barriers manifest across different audiences and to identify communication opportunities for addressing them (Kim & Grunig, 2011) (Figure 2). There are 2 key benefits to applying this theory to public engagement with biodiversity conservation. First, like the models described above, the theory conceptualizes individual motivation within the sociocultural context and considers the influence of public motivation and engagement. The model therefore encourages conservation organizations to consider different outcomes and levers to create change, such as social mobilization and advocacy, in addition to behavior change. The second benefit is the audience segmentation method the theory provides. The situational theory of problem solving conceptualizes issue engagement as a spectrum of active involvement with an issue. This spectrum allows for useful segmenting of the general public into different types of publics, which are more specific groups of individuals with defined relationships to the issue at hand. Situational motivation mediates engagement and represents “the extent to which a person stops to think about, is curious about, or wants more understanding of a problem” and is understood as an effect of problem recognition, involvement recognition, and constraint recognition (Kim & Grunig, 2011, p. 132). Considering whether these recognition factors are at high or low levels provides an audience segmentation method resulting in 4 public types: active, aware, latent, and nonpublic (Grunig & Hunt, 1984) (Figure 2).

Active publics are already taking action on the issue. They are highly engaged and may provide critical support enabling success (e.g., conservation volunteers). An aware public recognizes the issue of focus as a problem but is not taking action. Latent publics do not recognize the issue as a problem. This encompasses audiences who are unaware of or are opposed to conservation actions, including audiences who may undermine conservation success through disruptive behaviors, whether knowingly or unknowingly (e.g., allowing dogs off‐lead in protected areas) (Mengak et al., 2019; Selinske et al., 2020; Stern, 2000). The nonpublic is an audience for whom the issue is not directly relevant; knowing whom to exclude can be as important as whom to target. Identifying which public types are relevant for engagement assists audience targeting and saves time and resources. From a strategic communication perspective, the goal is to devise interventions that maximize behavior change, issue support and engagement, and ultimately increase the engaged public (e.g., by increasing issue relevance or empowering action).

### Our approach

To identify barriers relevant to public engagement with biodiversity, we drew on literature in conservation, behavioral science, strategic communication, and social change that related to interventions aiming to change the knowledge, perceptions, attitudes, or behavior of individuals for the purposes of environmental conservation. We then synthesized the barriers into 5 categories: lack of knowledge, misaligned values, low self‐efficacy, low personal or social relevance, and limiting structural context. In determining these categories, we considered the SEM levels, how barriers were likely to manifest across the 4 situational theory publics for biodiversity conservation issues, and the provision of a relevant and accessible discussion for conservation professionals. We examined these barrier categories to provide examples of how they manifest across different publics and identified communication approaches to address them (Figure 2; Table 1). Given many of these interventions are yet to be evaluated in the conservation context, it was not our intention to suggest that they are certain to overcome the barriers. Rather, we suggest they are promising options that can guide future research and practice.

**TABLE 1:** Examples of communication approaches and opportunities for overcoming barriers to engagement for each of 4 types (non, latent, aware, active)^a^ of public as defined by the situational theory of problem solving (Kim & Grunig, 2011).

Barrier	Non‐Public	Latent	Aware	Active
Lack of knowledge	OPPORTUNE EXPOSURE experience‐based learning; e.g., Arid Recovery reserve tours, Zoos Victoria animal encounters NUDGING target actions where knowledge of the issue is not a necessity; e.g., Zoos Victoria's Seal the Loop campaign installs bins specifically designed to contain fishing line at popular fishing sites in place of open bins	RAISE AWARENESS education programs or awareness campaigns; e.g., Zoos Victoria community conservation campaigns (Pearson et al., 2014) connect what the audience already knows to new information use a messenger or source trusted by the audience; e.g., using veterinarians as trusted messengers to encourage domestic cat containment (Ma & McLeod, 2023) present clear on‐ground implications or examples; even where knowledge is a primary barrier, audiences are unlikely to listen to or take on board information if it is not presented in an engaging way or related to their own experiences or understanding (e.g., Cotton et al., 2015; Nisbet & Scheufele, 2009; Reincke et al., 2020)	PROVIDE ACTIONS AND EMPOWER communicate a clear action individuals can take; e.g., Birdlife Australia's Birds in Backyards program present accessible opportunities to engage with information and ask questions; e.g., information booths at local markets	PROVIDE ACTIONS AND EMPOWER provide information on successful results or outcomes; e.g., member newsletters sharing updates on impact for those already taking action
Misaligned values		WORK WITH VALUES use a known and trusted messenger or find allies in target communities and support or elevate their work; e.g., using veterinarians as trusted messengers to encourage domestic cat containment (Ma & McLeod, 2023) connect preexisting values to issue; e.g., Zoos Victoria's Safe Cat, Safe Wildlife campaign reframes the issue to focus on keeping domestic cats safe inside, rather than framing cats as the enemy (i.e., feral cats destroying wildlife) make the intervention attractive and desirable; e.g., appeal to self‐interest, appeal to known values of target audience, provide compensation or incentives (MacFarlane et al., 2022) use a values‐based social norms approach; e.g., Harvard Alcohol Project to create social acceptability of the designated driver concept	REINFORCE VALUE ALIGNMENT AND RELEVANCE use message framing that elevates aligned values and avoid messaging that undermines them; e.g., focus on environmental or community framing rather than economic framing of issue (Kusmanoff, 2017; Marquina et al., 2022)	
	NUDGING target approaches and actions where value alignment is not a necessity; e.g., increasing regulation reminders to address compliance issues in recreational fishing (Mackay et al., 2018)			REENFORCE IDENTITY use identity messaging if an individual identifies as the kind of person who cares or acts for nature, reinforcing this identity can help trigger action and foster collective identity (Gulliver et al., 2021); e.g., “As someone who cares about nature, I focus on planting native plants in my garden,” “I love the outdoors, so I want to help protect it”
Low personal and social relevance	SOCIAL NORMS use social norming language; language that emphasises social norms can be highly effective at increasing conservation behavior intentions (Kusmanoff et al., 2020; Niemiec et al., 2020; Stern et al., 1999; Zhang et al., 2017); e.g., descriptive norms = perceptions of how common a behavior is (e.g., “We all recycle.”); injunctive and subjective norms = perceptions of whether others in the same social group or influential people think a behavior should be performed (e.g., “David Attenborough says we all should be taking better care of nature…”); personal norms = individual behavior standards flowing from personal values and identity (e.g., “Are you someone who cares about nature? If so, sign up for our mailing list.”)			
			REINFORCE VALUE ALIGNMENT AND RELEVANCE talk about the here and now; emphasizing when an issue is relevant locally and immediately (rather than in the future) makes it easier for audiences to see it as relevant to them (e.g., Bar‐Anan et al., 2006; Jones et al., 2017) make the action relevant to them and their social group; e.g., relevant to their locality or situation (engaging parents with actions they can feasibly engage their family in or using message appeals focused on preserving nature and wildlife for future generations) (e.g., Hogg & Reid, 2006) use messengers trusted by the audience; e.g., local community members, scientists, firemen, farmers	
Low self‐efficacy			PROVIDE ACTIONS AND EMPOWER make the action feel clear, easy, and achievable; e.g., Gardens for Wildlife provides information and support in the form of garden visits and expert advice; Zoos Victoria's Safe Cat, Safe Wildlife website provides cat hacks and expert advice to assist individuals in containing their cats (also see Bandura, 1977; Kim & Jang, 2018) provide positive feedback; e.g., certificates of achievement, thank‐you signage in parks provide pathways to mentorship and leadership, especially for ongoing actions; e.g., online community forums to connect and share advice, community groups	
				REENFORCE IDENTITY provide clear and easy ways to signal affiliation or leadership roles if an individual identifies as a part of a group who cares or acts for nature, they will continue to take action; providing skills to obtain in‐group status can be a motivating factor for action, e.g., friends of groups, advisory group membership
Limiting structural context	NUDGING create the right context for activating this audience or public to take the desired action; e.g., changes to infrastructure, law, or policy changes, choice architecture (i.e., nudging)		PROVIDE ACTIONS AND EMPOWER target audiences that can feasibly engage in the desired action ensure resources and infrastructure are available so the action is possible; e.g., appropriate bins available for disposal of plastic waste (Kusmanoff et al., 2022)	
				ADVOCACY work with active audiences to advocate for structural change; e.g., advocate for environmental policy change (Gulliver et al., 2022) or participate in new ways of measuring the value of nature (Pascual et al., 2023)

^a^ Defined in the “Situational theory of problem solving” section.

## BARRIERS TO ENGAGEMENT WITH CONSERVATION

### Lack of knowledge

Although knowledge is not sufficient on its own to motivate action, it is one of many factors that influence decision‐making and behavior change. It is therefore important to consider what an audience already knows about an issue (Groffman et al., 2010; McKenzie‐Mohr, 2011). Knowledge is key for individuals to be aware of an issue, understand the extent and impact of an issue, and know how to act on it (Wynveen & Sutton, 2016). For example, beach recreationists who are aware of migratory shorebirds and understand that disturbance negatively affects them will walk around shorebird flocks to minimize their disturbance of shorebirds when using the beach (Comber & Dayer, 2023).

An individual's state of knowledge can be influenced by the difficulty of understanding or uncertainty around interpreting complex issues, such as perceived conflicting scientific evidence or uncertainty around statistical trends (Lorenzoni et al., 2007). For example, the conflicting views on free‐ranging dogs in Australia and the different names used to identify them (*dingo* vs. *wild dog*) may trigger different knowledge frames, values, and norms around their conservation and control (Kreplins et al., 2018). Different worldviews and knowledge systems add further complexity to understanding and valuing nature and biodiversity (Pascual et al., 2023). Different ways of knowing (i.e., ontologies and epistemologies) may result in conflicts between what is accepted as legitimate knowledge or evidence or lead to miscommunication or misinterpretation about what the key issue is (e.g., threatened species recovery or overall ecosystem and community health) and what the appropriate solutions are (Barbour & Schlesinger, 2012; Kadykalo et al., 2021; Watson, 2013; Weiss et al., 2013). Understanding can also be influenced by cognitive biases, such as confirmation bias (i.e., “the seeking or interpreting of evidence in ways that are partial to existing beliefs, expectations, or a hypothesis in hand”), which may prevent individuals from accepting new knowledge that conflicts with their preexisting understanding (Nickerson, 1998, p. 1). Another bias, shifting baseline syndrome, describes how individuals may come to accept a situation simply because it has become the norm (Papworth et al., 2009; Poortinga et al., 2011). For example, forest logging that results in an altered grassland landscape over generations may result in perceptions that this altered landscape is the natural state.

**FIGURE 3:**
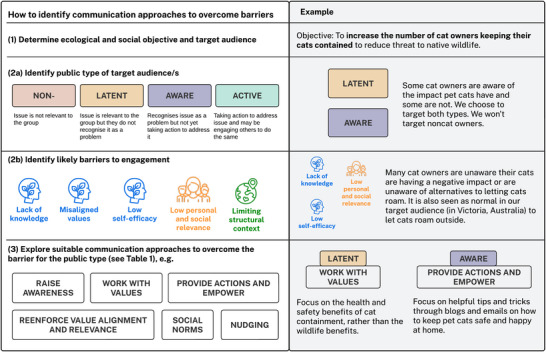
Approach to identification of potential communication approaches to overcome barriers for different public types. Example drawn from van Eeden et al. (2021) and Zoos Victoria's Safe Cat, Safe Wildlife campaign (safecat.org.au).

The communication opportunities for conveying, clarifying, and updating knowledge about biodiversity conservation depends on the relationship audiences have to biodiversity loss (Figures 2 & 3; Table 1). For active publics, opportunities include sharing success stories and recent updates, whereas for aware publics, information may focus on the actions that they can take. For latent publics, communication should focus on raising awareness using trusted messengers and ensuring alignment with their preexisting knowledge. For nonpublics, information by itself will not make an impact; however, raising the visibility of the issue in the public domain will help provide the context for them to shift to a more active role.

### Misaligned values

Individuals can be aware of an issue and yet not recognize it as a problem because it does not align with their values or expectations. Values are complex, interrelated beliefs that arise from knowledge, experiences, culture, and religion (Schwartz, 1992). Values and their resulting expectations can manifest on both personal and social levels. Misaligned values and expectations can manifest as priority differences, skepticism, shifting responsibility, apathetic disinterest, and self‐interest. Priority differences arise when biodiversity conservation is considered a low priority compared to other issues (e.g., humanitarian crises) (Uzzell, 2000) or when overshadowed by other priorities in daily life (e.g., health care) (Maslow, 1943). For example, communities living alongside large carnivores may be unwilling to conserve them if they pose a risk to life and property (Jacobson et al., 2012; Krafte Holland et al., 2018). Even when considered important, if biodiversity conservation is seen as a low‐priority issue, then an individual is unlikely to become engaged in probiodiversity behaviors (Schultz, 2011; Uzzell, 2000). Indeed, if values clash with an existing knowledge system or worldview, then antagonistic or skeptical positions can result.

Values can be influenced by exposure to messages or narratives through social or media discourse. The way messages are framed in the media can diagnose problems, place blame, propose solutions, and make a moral case for certain actions (Elliott, 2020; Entman, 1993). For example, much media representation of COVID‐19 framed bats as the cause of the disease, reinforcing negative associations between wildlife and zoonoses (Gregg et al., 2021; MacFarlane & Rocha, 2020). Indeed, framing the issue instead in a way that highlighted both human and animal causes elicited greater support for proconservation policies (Shreedhar & Mourato, 2020). Improved understanding of how different values are used when framing information is useful for conservation, particularly when it comes to partnerships or conflicts with industries, such as mining, agriculture, or housing construction. This is particularly crucial in the current social and political climate, in which vocal minorities are readily activated against issues that are perceived to be part of a broader culture war, making careful use or avoidance of message frames important in avoiding potential backlash. For example, the Great Northern Brewing Company received backlash when they partnered with the Foundation for National Parks and Wildlife to raise money to create more national parks (see https://cub.com.au/statement‐from‐great‐northern/). This was labeled as woke by vocal advocates, who wanted to save the bush by keeping parks open for recreational use (e.g., hunting, fishing) and so were opposed to these areas becoming national parks. From a segmentation perspective, this is an example of misunderstanding what the audience cares about. In this case, the audience did not necessarily care about the well‐being of nature, but rather access to it.

It is only to be expected that individuals are likely to act in a way that provides them with the most benefits and least costs, regardless of the environmental impact (de Groot & Steg, 2009). For biodiversity conservation, this is particularly relevant because many probiodiversity behaviors will not directly benefit the individual who undertakes them (Selinske et al., 2018). Indeed, probiodiversity action may be to their own relative detriment (e.g., by paying more for an alternative product) and be inherently reliant on the audience's altruistic and probiodiversity values (de Groot & Steg, 2009; Winkler‐Schor et al., 2020). Where an action is out of step with social norms, there may also be perceived social costs due to nonconformity (Telesetsky, 2017). Some individuals may therefore shift the responsibility toward others, particularly government and other organizations with greater perceived responsibility and resource capacity (Bickerstaff, 2008; Bosone et al., 2022). Individuals may also contend that human technology will eventually be able to resolve environmental crises (Stoll‐Kleemann, 2001) or invoke the status quo, favoring doing things the way they have always been done (Telesetsky, 2017). Detrimental behaviors may also be driven by a fear that one will miss out on benefits that others will continue to gain (i.e., free‐rider effect) (Lorenzoni et al., 2007). This is particularly problematic as most conservation issues are large‐scale problems that require collective community participation to achieve meaningful results (Stern, 2000). The desire to retain or improve one's own lifestyle or status can reduce common motivators for conservation behaviors, such as empathy, communality, and altruism (de Groot & Steg, 2009). In contrast, there are successful examples of social status playing a key role in motivating some select biodiversity conservation behaviors (e.g., private land conservation; Selinske et al., 2015).

Overall, values‐based communication opportunities are most relevant for latent and aware publics (Table 1). There are many opportunities to promote probiodiversity behaviors without having the audience highly value nature (e.g., eating less meat because it is cheaper and healthier, using car share schemes because it is cheap and convenient, buying electric vehicles because you do not pay for petrol). For latent publics, therefore, a key opportunity is to highlight values they already hold that align with probiodiversity actions, including using trusted messengers and messages that highlight potential benefits for individuals and their communities, and reinforce social norms. Setting clear expectations and being consistent in messaging are important for aware and active publics to maintain trust and keep them engaged. For active publics, reinforcing their identity can help them continue to feel connected and maintain trust that their efforts make a difference.

### Low personal and social relevance

A strong and personally meaningful connection between an individual and an issue is a great motivator and a conducive context for action (Kim & Grunig, 2011). Meaningfulness can be inwardly personal or related to social connections. Renninger and Hidi (2015) distinguish between individual interest, which is a predisposition to re‐engage with a particular activity or topic over time, and situational relevance, which is sparked by factors in the environment. Perceived relevance can lead to more meaningful engagement and to longer term individual interest. In biodiversity conservation, many factors can decrease personal and social relevance of conservation, including psychological distance, intangibility, and social norms (Bar‐Anan et al., 2006).

The concept of psychological distance proposes that the way in which an individual construes an issue depends on whether that issue is perceived as being near or far on multiple dimensions (Trope & Liberman, 2010; Wang et al., 2021). Geographic or spatial psychological distance refers to the physical distance in space from a problem and can include the physical separation of people from nature (Schultz, 2011) and interactions with species, as well as the perception that an issue is taking place far away (McDonald et al., 2015). Temporal psychological distance describes the perception that an issue will occur far into the future and may be associated with a propensity for self‐interest despite facing a future of diminishing resources (McDonald et al., 2015; Zhang et al., 2019). With decreased experience of nature and biodiversity, the relationship between individuals and biodiversity loss is abstract and feels far away (Pett et al., 2016; Wang et al., 2021). This extinction of experience (Miller, 2005; Pyle, 1978) is thought to contribute to increasing apathy and a declining sense of care and responsibility toward the natural environment (Gaston & Soga, 2020). Even researchers working directly with nearly extinct species will rarely witness dramatic species decline or extinction directly (but see Lunney et al. [2011]). This intangibility (abstractness) and psychological distance may contribute to increasing apathy and lead individuals to only act on issues they view as close to home. Audiences may struggle to empathize with species that appear very different to themselves, particularly those that are feared (e.g., snakes), perceived as unappealing (e.g., insects), misunderstood (e.g., native rodents), rarely seen (e.g., frogs), or easily overlooked (e.g., plants, marine life) (Balding & Williams, 2016; Batt, 2009; Troudet et al., 2017). In contrast, common flagship species that are seen as appealing, familiar, or symbolically resonant, such as tigers, koalas, and butterflies, benefit from their social relevance (Bowen‐Jones & Entwistle, 2002; Clucas et al., 2008).

Social relevance emerges from a perceived connection or relevance to others (Cialdini & Goldstein, 2004; Hogg & Reid, 2006). Social norms are “informal and shared behavioral rules that prescribe what one ought or ought not to do that people comply with because of social expectations and potential social sanctions” (Andrighetto & Vriens, 2022, p. 3). The behaviors of individuals will influence their peers, particularly through social‐signaling actions (e.g., sharing social media posts) that hold potential to influence social norms by providing positive (or negative) reinforcement for behaviors (Amel et al., 2017; Naito et al., 2022; Selinske et al., 2020). Some individuals and groups will have a greater social influence than others due to their proximity to the individual or perceived role as leaders (Hogg & Reid, 2006). Where probiodiversity behavior requires deviation from prevailing social norms, these norms present a barrier to biodiversity conservation buy‐in and behavior (Wynveen & Sutton, 2016). However, this desire to fit in means that social praise can be a strong motivator for behavior change (Handgraaf et al., 2013). The language used to talk about biodiversity in social and media discourse can create or reinforce message frames and narratives that influence broader public understanding of the personal and social relevance of biodiversity conservation as an issue (e.g., crisis or anthropocentric narratives) (Gregg et al., 2021; Louder & Wyborn, 2020).

Communication opportunities for targeting personal and social relevance of biodiversity conservation depend on the audience's level of involvement (Figure 1; Table 1). For latent publics with no existing level of involvement with biodiversity loss, the opportunity is to demonstrate the personal relevance that they do not yet see, making it concrete and as local as possible. Similarly, aware publics can be motivated into deeper involvement by conveying the personal and social impact of the problem and creating situations for specific action and engagement (i.e., social mobilization). Highlighting social norms and opportunities for social mobilization and collective action are ways to create social relevance, especially for nonpublics for whom there is no personal relevance or involvement. The actions of proactive organizations and governments can have a strong positive influence, even when only rhetorical or symbolic, and convey a sense of importance or relevance to an issue. Social movement calls from advocacy organizations for collective action can be effective by perpetuating new social norms and providing individuals with support and motivation through their peers (Johnson, 2012). However, this relies on individuals trusting the initial source of the call and having faith that the collective action will be effective (Cotton et al., 2015; Le et al., 2015).

### Low self‐efficacy

Where interventions aim to promote specific behavior change, enhancing self‐efficacy is key for promoting action (Bandura, 1977; Clayton et al., 2017; Tabernero & Hernández, 2010). Self‐efficacy is one's individual belief in one's ability to execute behaviors to get a particular result, and response efficacy is one's belief in the effectiveness of the action (Bandura, 1977). We considered both here. Our focus is on the perceptions of individuals as to their own ability to engage; their actual ability is also influenced by contextual factors, which are further discussed in “Limiting structural context” below.

Biodiversity conservation can be perceived as such a large and complex issue that individuals may feel their contribution will not make a difference (Bandura, 1977; Hunter & Jordan, 2020). Fatalism, the belief that a certain outcome is inevitable regardless of an individual's actions, has been particularly problematic relative to the issue of climate change (Lorenzoni et al., 2007). A fatalistic audience may care about an issue but be passive and despondent when it comes to engaging in positive behavior. For example, individuals presented with information about an overwhelming rate of extinction may think it inconceivable that they could possibly make a meaningful difference. Fatalism can be exacerbated by oversaturation of messages, particularly negative messages or messages that include appeals for money or time (Sherry, 2002). If individuals receive constant requests for care or action, they may perceive this as an indication of an enormous issue that they cannot hope to fix alone (similar to the behavioural economics concept of “mental bandwidth” [Samson, 2021]).

The perceived difficulty of a specific action can have a strong impact on whether an individual is motivated to complete it. An action may be perceived to be difficult if it presents risks or costs, such as time, money, status, or other social or physical costs (particularly friction costs, i.e., the hassle factor). Individuals tend to be risk averse, particularly when they are unsure about an issue, so a costly behavior can dramatically decrease the motivation to act (Litchfield et al., 2018). Costs are not always material, and the willingness of individuals to act may be influenced by how they anticipate they may feel after an action (e.g., pride, removal of ecoguilt, embarrassment due to engaging in behaviors outside social norm) (Graton et al., 2016; Han & Hyun, 2016). Despite high motivation, if an action requires a break in habit, the perceived difficulty of the action may prove greater than otherwise expected (Klöckner & Verplanken, 2018). Even actively engaged individuals may lose motivation to continue if there are insufficient positive feedback mechanisms following an enacted behavior (e.g., encouragement or rewards).

Communication opportunities that build capacity and increase self‐efficacy will have the greatest impact on aware and active publics (Figure 1; Table 1). For active publics, the unique opportunities include developing advocacy skills and empowering individuals to engage others to do the same. Both active and aware publics can be empowered to develop new skills and practice actions that overcome constraints they recognize at their individual and system levels. Consistency and clarity of the actions provided will build trust and increase self‐efficacy. For non‐ and latent publics, low‐self efficacy by itself will not have an impact; however, providing clear, simple actions that can be taken by anyone will build capability and lower this potential barrier to engagement.

### Limiting structural context

All the above barriers are influenced by an individual's personal and social context, including social, cultural, and structural (e.g., infrastructure) elements (Stern, 2005). For example, in areas with insufficient or sparsely distributed rubbish bins, individuals may be physically prevented from being able to dispose of waste and therefore limited in their capacity to address associated problems, such as impacts of plastic pollution on local waterways and wildlife (Yukalang et al., 2017). Other contextual limitations on an individual's engagement can include socioeconomic circumstances, governmental structures, laws and regulations, a lack of technology or enabling initiatives, current political or cultural governance, or other cultural and social factors (Schmitt et al., 2020; Semenza et al., 2008). Communication approaches can be applied to implement advocacy and social mobilization or collective action campaigns and to affect such actions over time. However, it is also necessary to consider these barriers when selecting appropriate objectives and audiences. For example, it may be completely inappropriate (and ineffective) to design communications asking for large donations from households in low‐socioeconomic suburbs or for volunteer time from busy parents (Gregg et al., 2022). Addressing structural barriers to biodiversity conservation through transformative change (e.g., policy change) is crucial to addressing the biodiversity crisis on national and global levels (Jones & Niemiec, 2023; Pascual et al., 2023; Waddock, 2020). Promoting high engagement with biodiversity conservation is a key part of ensuring the cultural context and social will required for this transformative change.

Although most conservation professionals will not be able to directly overcome the barriers resulting from limiting structural context, understanding these barriers is key to recognizing opportunities for impact through social, institutional, cultural, and organizational levers. For nonpublics and latent publics, structural and social nudges (e.g., curbside recycling) provide the greatest opportunity to support probiodiversity actions. Providing ways and means for aware and active publics to participate and empowering them to engage in socioculturally appropriate ways are key. Also important is empowering active publics to engage in advocacy to help change the systems that generate these structural barriers.

## CONCLUSION

There are many well‐established strategic approaches to public engagement that show promise for biodiversity conservation (Gregg et al., 2021; Kidd & Dayer, 2020; Smith et al., 2024; Thomas‐Walters et al., 2022). As interest in such interventions for conservation increases, targeted research is required to test the effectiveness of these interventions (Selinske et al., 2021). Drawing on preexisting evidence and experience inside and outside the conservation field will be key to success in this endeavor because the processes that shape individuals’ attitudes and behaviors are nuanced and complex (Kidd et al., 2019). We have introduced readers to this complexity by drawing from systems theory, the situational theory of problem solving, and a broad multidisciplinary literature. The frameworks and discussion we presented provide an accessible starting point for conservation professionals to explore and identify relevant audiences and barriers for their specific conservation challenges and inform the planning and design of interventions. To successfully engage audiences with biodiversity conservation, ongoing research is required to empirically test the suitability of different approaches to overcome barriers in different publics and contexts. Increased focus is also needed on exploring pathways for shifting audiences to be more engaged and active in biodiversity conservation as a broad issue. We hope our discussion provides a starting point for this challenging but necessary endeavor. Although barriers to engagement present a challenge to conservation professionals, they also provide an opportunity to plan, design, and test new interventions to improve public engagement with biodiversity and ultimately work toward long‐term success for conservation.
